# Xylooligosaccharides from Barley Malt Residue Produced by Microwave-Assisted Enzymatic Hydrolysis and Their Potential Uses as Prebiotics

**DOI:** 10.3390/plants14050769

**Published:** 2025-03-03

**Authors:** Shah Zaib Fareed, Pipat Tangjaidee, Tabkrich Khumsap, Wannaporn Klangpetch, Suphat Phongthai, Apinun Kanpiengjai, Chartchai Khanongnuch, Kridsada Unban

**Affiliations:** 1 Master’s Degree Program in Food Science and Technology (International Program), Faculty of Agro-Industry, Chiang Mai University, Chiang Mai 50100, Thailand; shahzaibfareed2001@gmail.com; 2 Division of Food Science and Technology, Faculty of Agro-Industry, Chiang Mai University, Chiang Mai 50200, Thailand; pipat.t@cmu.ac.th (P.T.); tabkrich.khumsap@cmu.ac.th (T.K.); wannaporn.u@cmu.ac.th (W.K.); suphat.phongthai@cmu.ac.th (S.P.); 3 Division of Biochemistry and Biochemical Innovation, Department of Chemistry, Faculty of Science, Chiang Mai University, Chiang Mai 50200, Thailand; apinun.k@cmu.ac.th; 4 Research Center for Multidisciplinary Approaches to Miang, Multidisciplinary Research Institute (MDRI), Chiang Mai University, Chiang Mai 50200, Thailand; chartchai.k@cmu.ac.th; 5 Department of Biology, Faculty of Science, Chiang Mai University, Chiang Mai 50200, Thailand; 6 Research Center of Microbial Diversity and Sustainable Utilization, Chiang Mai University, Chiang Mai 50200, Thailand

**Keywords:** barley malt residue, xylooligosaccharide, microwave-assisted enzymatic hydrolysis, prebiotics

## Abstract

Barley malt residue (BMR) was subjected to microwave-assisted enzymatic hydrolysis to evaluate its potential as a raw material to produce xylooligosaccharides (XOS) suitable for use as a prebiotic. The influent factors on XOS production, microwave power, exposure time, and xylanase dosage were ascertained with response surface methodology based on Box–Behnken design (BBD). The fitted models of XOS and xylose yields were in good agreement with the experimental results. Using a microwave power of 1235.1 W, a 6 min exposure time, and a xylanase concentration of 89.12 U/g substrate gave the highest yield of XOS: 208.05 mg/g substrate at 4 h of enzyme incubation time. Based on the product composition, BMR-XOS purification by *Saccharomyces cerevisiae* treatment was superior to the process of activated carbon adsorption and ethanol precipitation treatment and was selected for further experiments. Thin-Layer Chromatography (TLC) and high-performance liquid chromatography (HPLC) clearly elucidated the oligosaccharide compositions, and the result of Fourier Transform Infrared Spectroscopy (FTIR) confirms the molecular structure and sugar components of achieved BMR-XOS. In vitro fermentation of BMR-XOS obtained from this study by the selected probiotics, *Lactococcus lactis* TISTR 1401, *Levicaseibacillus brevis* FS 2.1, *Lactobacillus casei* TISTR 1463, showed similar prebiotic activity compared with the commercial XOS, galactooligosaccharides (GOS), xylose, and glucose (control). In conclusion, the present study was successful in establishing the use of barley malt residue for the extraction of xylan and XOS, which could be further used as a prebiotic.

## 1. Introduction

Barley malt residue (BMR) is a major byproduct of the beer fermentation process, constituting up to 85% of brewery solid waste, with around 39 million tons produced annually [1]. Due to its low economic value, recent studies have focused on converting BMR into higher-value food products with health benefits [2], due to its high fiber content and specific ability to stimulate the growth of beneficial gut bacteria [3]. BMR contains 5.8–6.0% lipids, 15.3–16.2% protein, 6.4–7.0% moisture, and 46% dietary fiber, including lignin, cellulose, arabinoxylan, and mainly xylan, which is a promising feedstock for producing XOS with prebiotic activity [4,5,6]. Prebiotics are defined as non-digestible oligosaccharides that promote the growth of beneficial microorganisms in the human/animal gastrointestinal tract [7]. The International Association for Scientific Prebiotics and Probiotics (ISAPP) defines prebiotics as substrates selectively utilized by host microorganisms to confer health benefits. Prebiotics are widely used for improving gastrointestinal, cardiovascular, neurological, and other systemic health issues [8]. Xylooligosaccharides, consisting of 2–10 xylose units linked by β-1,4 bonds, are heat-resistant, acid-stable, and emerging prebiotics [9]. Xylooligosaccharides exhibit significant potential for practical applications across various sectors, including pharmaceuticals, animal feed formulations, and agriculture. Notably, their most substantial market expansion was within food-related industries. In this domain, XOS offers advantages over other oligosaccharides concerning health benefits and effective concentration thresholds. However, their relatively high production costs, ranging from USD 25 to 50 per kilogram, have impeded broader and more rapid market adoption [10,11]. Therefore, finding a low-cost XOS-producing process is necessary for industrialization. Various methods such as autohydrolysis, acid hydrolysis, alkali treatment, and microwave pretreatments are used in the processes to achieve XOS from a variety of natural or agricultural sources including tobacco stalk, barley straw, and wheat bran [12,13,14,15], with microwave-assisted enzymatic hydrolysis proving particularly effective for enhancing XOS yield results in reduced energy consumption, lower equipment requirements, and shorter processing times compared with traditional methods [15,16,17], leading to the cost reduction process of XOS.

Microwave-assisted extraction efficiently facilitates material extraction by directly heating solvents and interacting with water molecules in plant tissues, causing structural rupture and releasing active compounds [18]. Unlike acid hydrolysis, which generates toxic byproducts, enzymatic methods avoid harmful effects and require less equipment [19]. Traditional extraction methods like fermentation are time-consuming and produce significant chemical waste [20,21]. Microwave-assisted extraction offers a faster, sustainable alternative, requiring minimal solvent and enhancing enzymatic activity, resulting in higher yields of fermentable sugars and valuable compounds from lignocellulosic biomass [22]. From previous reports, microwave-assisted extraction reduces extraction time, boosts yield, minimizes solvent use, and enhances enzymatic activity, aligning with green chemistry principles. Studies confirm its ability to increase the XOS/xylose ratio which is more effective for promoting probiotic growth making the achieved XOS-producing process an effective method for broader industrial applications [16,23]. From the previously reported, microwave pretreatment at 170 °C for 2 min effectively enhanced the enzymatic production of soluble glucan [24]. Furthermore, microwave treatment at 200 °C for 5 min significantly improved the extraction yield of oligosaccharides from spruce wood [25]. This technology aligns with sustainability goals, turning waste into functional food ingredients that support gut health, which affects the gut microbiome by increasing the beneficial bacteria and short-chain fatty acid production [26,27,28,29].

The present study aimed to develop a technique to produce XOS from BMR using microwave-assisted enzymatic hydrolysis, a green technology that enhances process efficiency, reduces chemical waste, and improves enzymatic activity. As far as we know, most oligosaccharide production from BMR was derived from the extracted xylan [17], and there are no studies specifically focused on the direct production and purification of XOS from BMR without conventional pretreatment processes. This work describes an optimization study of XOS production from BMR using microwave-assisted enzymatic hydrolysis. The purification of XOS was achieved through treatments with *Saccharomyces cerevisiae*, activated carbon, and ethanol. The extracted XOS was also characterized and the prebiotic potential of XOS was assessed through fermentation studies with the probiotic strains.

## 2. Materials and Methods

### 2.1. Materials

Barley malt residue (BMR) was obtained from a local brewery industry (Suthep, Mueang, Chiang Mai, Thailand). The BMR residue was in a wet form with a moisture content of about 80% and was dried in a hot-air oven at 50 °C until a constant weight was attained to reach a moisture content lower than 10% (*w*/*w*), and then crushed with a blender and sieved through a 20-mesh screen. Barley malt residue powder was stored in a vacuum-sealed aluminum bag at room temperature until further usage. Commercial xylanase (1500 U/mL) was purchased from Value Industrial Products Co., Ltd., Bangkok, Thailand. A mixture of xylose (X1), xylobiose (X2), xylotriose (X3), xylotetraose (X4), xylopentaose (X5), and arabinose was used as the standard for the determination of oligosaccharides. Commercial prebiotics including commercial GOS, inulin, and XOS were purchased from Wako Pure Chemical Industries (Tokyo, Japan) to compare prebiotic properties with the obtained oligosaccharides.

### 2.2. Compositional Analysis of Barley Malt Residue

The physicochemical parameters of BMR, including moisture content, ash content, lipid content (measured by the Soxhlet method), and protein content (determined by the Kjeldahl method), were analyzed using the Association of Official Analytical Chemists [30]. The samples were also analyzed for their amounts of lignin (TAPPI T222-om-02, [31]), cellulose (TAPPI T203-om-93, [32]), and holocellulose [33], according to the methods of the Technical Association of the Pulp and Paper Industry (TAPPI).

### 2.3. Statistical Optimization of Oligosaccharides Production Through Microwave-Assisted Enzymatic Hydrolysis of Barley Malt Residue

The amount of 2 g dried and powdered BMR was mixed with 10 mL of deionized water in a test tube. A closed-vessel microwave digestion system ETHOS 1600 was used for sample pretreatment, which consisted of a 16-position rotor with an easyTEMP contactless sensor, using modified polytetrafluoroethylene (PTFE) vessels of 100 mL volume. Each vessel contained 45 mL of sample, and 16 vessels were processed at the same time. Specifically, 2 g of extractive-free BMR was suspended in 10 mL of distilled water and heated at 1200 W, 30 bar pressure, for 4 min, followed by 5 min of cooling. Temperature and pressure sensors monitored the process. The production conditions of xylooligosaccharides were studied using a Box–Behnken design (BBD) experiment using three variables: microwave power (1000–1400 W), exposure time (2–6 min), and the concentration of xylanase enzyme (20–100 U/g substrate) by setting 3 levels +1, 0, and −1, as shown in Table 1, and designing 17 treatments, as shown in Table 2. The optimization study was designed using Design Expert software version 6.0.10. The samples were incubated at 50 °C and 200 rpm for 24 h. The reaction was terminated by immersion in boiling water for 5 min, followed by centrifugation at 12,000× *g* for 10 min. The XOS from BMR were analyzed by high-performance liquid chromatography (HPLC).

### 2.4. Analysis of Xylooligosaccharides

Xylooligosaccharides from BMR were analyzed by high-performance liquid chromatography (HPLC) using an ion-exchange column (Bio-Rad Aminex HPX-87H, 300 × 7.8 mm) (Hitachi HPLC series L, Tokyo, Japan). The analysis was performed with 0.005 M H_2_SO_4_ as the mobile phase at a column temperature of 50 °C and a flow rate of 0.5 mL/min (total time 30 min), with an RI detector. The diluted samples were filtered using a nylon filter 0.22 μm (Whatman GmbH, Dassel, Germany) before injection in the HPLC system. Standards included xylose (X1), xylobiose (X2), xylotriose (X3), xylotetraose (X4), xylopentaose (X5), and arabinose.

### 2.5. Microstructural Analysis of BMR Before and After Microwave Pretreatment

The surface morphology of BMR was investigated with a scanning electron microscope (SEM, JSM-IT200, JEOL Ltd., Tokyo, Japan) at various stages: untreated, post-microwave pretreatment, and post-enzymatic hydrolysis. Sample preparation included drying at 50 °C for 24 h, grinding into fine powder, mounting on specimen stubs, and sputter-coating with copper and was performed following Thipchai et al. [34]. Imaging was performed at an acceleration voltage of 10 kV and a magnification of 5000–15,000×. A beam spot diameter of 1 μm was maintained.

### 2.6. Purification of Xylooligosaccharide from Barley Malt Residue

After enzymatic hydrolysis, the supernatant was collected by centrifugation at 12,000× *g* for 10 min. The comparison of the following three methods was investigated for XOS purification: (1) ethanol precipitation, (2) activated carbon adsorption, and (3) microbiological treatment (*Saccharomyces cerevisiae*).

#### 2.6.1. Ethanol Precipitation Method

The volume of 50 mL liquid product was mixed with 150 mL of 95% ethanol in order to separate XOS from soluble impurities. The pellets formed were removed by centrifugation at 3000× *g* for 10 min. The supernatant containing targeted XOS was evaporated using a rotary evaporator (N-1000 Eyela Rotary Evaporator; Tokyo, Japan) at 40 °C and freeze-dried by lyophilizer. The lyophilized powder was reconstituted in water for subsequent analysis through TLC and HPLC analysis.

#### 2.6.2. Activated Carbon Adsorption Method

Activated carbon powder was introduced to the supernatant liquid with three loadings: 1, 5, and 10% (*w*/*v*). All varied activated carbon-mixed samples were homogenized at room temperature (25–30 °C) and placed on a rotary shaker at 200 rpm for 30 min to saturate and stabilize carbon carbohydrate adsorption. These mixtures were suction-filtered with a 20 mL Pyrex crucible filter and then washed using 4 × 50 mL of distilled and deionized water. The XOS-enriched carbon cake was thus obtained and eluted with 50% ethanol twice to recover the XOS of interest, as reported by Zhu et al. [35]. The liquid was evaporated using a rotary evaporator to remove ethanol, the concentrate was lyophilized and redissolved in water for TLC and HPLC analysis.

#### 2.6.3. *Saccharomyces cerevisiae* Treatment

*Saccharomyces cerevisiae* TISTR 5088 was applied to a modified method described by Cunha et al. [36] in order to purify XOS from BMR liquor obtained after microwave-assisted enzymatic hydrolysis. Yeast was grown in 3 mL of YM media (yeast extract, 3.0 g/L malt extract, 3.0 g/L peptone, 5.0 g/L glucose, 10.0 g/L) and incubated at 30 °C for 24 h. After that, the culture broth of yeast cells (around 10^6^ CFU/mL) was centrifuged at 4000× *g* 4 °C for 5 min and washed with 0.85% NaCl solution. Then, wet cell yeast was resuspended in 150 mL of BMR liquor and incubated at 30 °C for 24 h. The mixture was centrifuged at 4000× *g*, 4 °C, for 5 min. The yeast-treated sample was used for TLC and HPLC analysis.

### 2.7. Characterization of Xylooligosaccharide from Barley Malt Residue

#### 2.7.1. Thin-Layer Chromatography

Thin-Layer Chromatography (TLC) was used to elucidate the sugar profile, using the procedure described by [37] with slight modifications. The sample was spotted on an aluminum silica gel plate and dried. The plate was twice developed in a chamber saturated with *n*-butanol/ethanol/water (5.5:3:1.5 *v*/*v*/*v*) to increase XOS and xylose separation. Visualization was performed with 0.5% (*w*/*v*) thymol in 5% (*v*/*v*) H_2_SO_4_-ethanol and heating at 100 °C for 10 min. Megazyme commercial standards for xylose (X1), xylobiose (X2), xylotriose (X3), xylotetraose (X4), xylopentaose (X5), and arabinose were analyzed simultaneously on the TLC plate along with the sample.

#### 2.7.2. Fourier Transform Infrared Analysis

Fourier Transform Infrared (FTIR) spectroscopy was used to examine the molecular structure and functional groups of xylan and XOS obtained from barley malt residue. Spectroscopic characterization was carried out with an FTIR JASCO infrared spectrophotometer within the spectral range of 3600–400 cm^−1^ at a resolution of 2 cm^−1^ with eight accumulated scans. BMR residues were pretreated with spectroscopic-grade KBr and compared with xylose, commercial XOS, inulin, and GOS before analysis. Literature protocols for interpreting the spectral bands have been well established by Adapa et al. [38].

### 2.8. In Vitro Fermentation of Xylooligosaccharide from Barley Malt Residue by Probiotics

The probiotic lactic acid bacterial strains were used to study prebiotic activity in this experiment. *Lactococcus lactis* TISTR 1401 and *Lactobacillus casei* TISTR 1463 were purchased from the Thailand Institute of Scientific and Technological Research (TISTR) while the XOS-fermenting probiotic, *Levicaseibacillus brevis* FS2.1 was kindly provided by Dr. Apinun Kanpiengjai at Chiang Mai University [37]. These strains were grown on De Man, Rogosa, and Sharpe (MRS) broth under an aerobic environment prior to fermentation. The fermentation medium was composed of 10 g of different carbon sources, such as glucose, xylose, GOS, inulin, commercial XOS, and XOS from BMR. The medium consisted of 10 g casein peptone, 10 g beef extract, 5 g yeast extract, 2 g sodium acetate trihydrate (CH_3_COONa·3H_2_O), 2 g di-ammonium hydrogen citrate ((NH_4_)_2_HC_6_H_5_O_7_), 0.2 g K_2_HPO_4_, 0.2 g MgSO_4_·7H_2_O, and Tween 80. The ingredients were dissolved in water, and the final volume was adjusted to 1000 mL, followed by adding bromocresol purple to a final concentration of 125 ppm. The pH of the medium was adjusted to 6.5 by using 1 M NaOH or 1 M HCl and sterilized by autoclaving at 121 °C for 15 min. The bacterial strains were precultured in MRS broth at 37 °C for 24 h. After preculturing, 1% (*v*/*v*) of each bacterial seed culture was then transferred to the fermentation medium. The samples were periodically taken (0, 6, 12, 24, 36, and 48 h) to monitor the growth of the probiotic cultures in XOS from BMR and other carbon sources by measuring the optical density at 600 nm, pH, and microbial counts on MRS agar with logCFU/mL.

### 2.9. Analytical Methods

All experiments were performed in triplicate. The results are presented as values of the mean ± standard deviation. Statistical analyses were carried out using SPSS 11 software. Duncan’s one-way multiple comparisons were performed to determine significant differences (*p* < 0.05).

## 3. Results and Discussion

### 3.1. Composition of Barley Malt Residue

Barley malt residue, a common agricultural residue, poses environmental concerns when dumped or burned. However, it can be converted into a valuable prebiotic component, xylooligosaccharides. Plant biomass, including BMR, primarily consists of cellulose, hemicellulose, and lignin. The BMR used in this work had the following average composition (expressed as g per 100 g of dry matter) as shown in Table 3. The proximate analysis of BMR was carried out using the Association of Analytical Chemists (AOAC) standard procedure. This chemical composition is in good agreement with other values found in the literature for this feedstock material, which typically contains 19.2 to 40% hemicellulose, 12 to 33% cellulose, and 14.2 to 26.7% protein. Additionally, the studies have noted similar ranges for lignin (11.5–22%) and ash content (1.1–4.6%) [39,40]. Moreover, regarding the hemicellulose content, the BMR showed a value of 17.40% (Table 3), lower than that found by Meneses et al. [41] and Kanauchi et al. [42], who observed 19.2 and 21.8%, respectively. The lower hemicellulose content compared with previous studies could be attributed to variability in BMR composition, which is influenced by factors such as malt type, brewing conditions, and processing methods [43,44]. Even though a lower hemicellulose content might reduce the theoretical yield of XOS, the practical yield of XOS might be improved by the specific extraction process. Arabinoxylan is the primary constituent of the hemicellulose fraction of BMR, which can account for up to 25% on a dry weight basis [1]. The breakdown of arabinoxylan yields XOS with varying degrees of polymerization. XOS obtained by enzymatic treatment of wheat arabinoxylan and rice husk has been reported to have prebiotic potential [16,45]. It has been reported that BMR contains xylan around 14% of dry solid [46], which corresponds with hemicellulose content from our report, which is approximately 17.4% (Table 3). Additionally, xylans from other agricultural residues such as tobacco stalk, cotton stalk, sunflower stalk, and wheat straw were around 19–21% [47]. However, the chemical composition of BMR may vary depending on the brewery’s conditions and the ingredients used for brewing.

### 3.2. Optimization of Oligosaccharides Production Using Box-Behnken Design

The production conditions of XOS from barley malt residue were investigated using a Box–Behnken design (BBD) with three variables: microwave power, exposure time, and xylanase enzyme concentration. After 12 h of enzymatic incubation, a significant production of XOS was observed across most experimental conditions. As presented in Table 4, the yield of XOS begins to decline after 12 h of enzymatic fermentation. This reduction is hypothesized to result from the degradation of oligosaccharides into monosaccharides between 24 and 48 h. Xylooligosaccharide production from agricultural waste, such as cotton stalks and sugarcane bagasse, has been studied, with optimal conditions for enzymatic hydrolysis determined [48,49]. The yield of XOS typically peaks around 12 h of hydrolysis, with a maximum yield of 31.8% reported for sugarcane bagasse [49]. However, prolonged hydrolysis can lead to a decrease in XOS yield due to the conversion of oligosaccharides into monosaccharides by enzymes like β-xylosidase [50]. Analysis of reducing sugar and total sugar levels across the experiments indicated a consistent increase in reducing sugar content across all treatments, whereas the total sugar content remained relatively stable beyond the 12 h mark (Figure S1). Furthermore, statistical evaluation employing a second-order multiple linear regression model, as detailed in Table 5, corroborates these experimental observations.

From Table 5, the analysis of variance (ANOVA) demonstrates the significant effects of the independent variables (microwave power, exposure time, and enzyme concentration) on the XOS yield (dependent variable) using barley malt residue, with a confidence level exceeding 95% at 4 and 12 h. Regarding the F-value (<0.0001) of quadratic parameters obtained from fit summary analysis by the Design-Expert 6.0.10 program, the statistical modeling revealed that the quadratic model was the most suitable for predicting the optimal conditions for xylooligosaccharide production. The relationship was expressed mathematically through the derived equation.

Xylooligosaccharide content (mg/g substrate) in 4 h incubation.

XOS (mg/g) = +164.05 − 6.41A + 26.01B + 0.084C − 25.55A^2^ + 20.14B^2^ − 26.04C^2^ + 8.96AB − 2.06AC + 16.75BC (1)

Xylooligosaccharide content (mg/g substrate) in 12 h incubation.

XOS (mg/g) = +136.75 + 1.39A + 12.72B − 3.85C − 14.35A^2^ + 12.52B^2^ + 0.19C^2^ + 8.81AB − 0.45AC + 16.54BC (2)

where A = microwave power (watts), B = exposure time (min), and C = enzyme dosage (U/g substrate).

**Figure 1:**
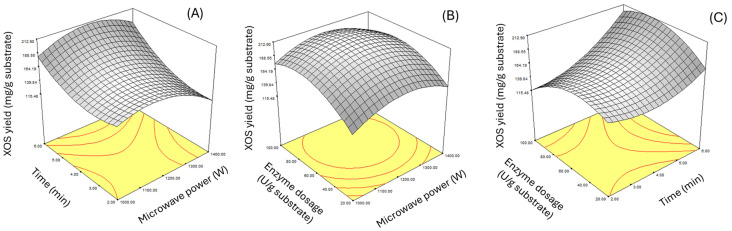
Response surface plot of xylooligosaccharide production from barley malt residue after 4 h enzyme incubation, showing the relationship between microwave power and exposure time (**A**), microwave power and xylanase enzyme concentration (**B**), and xylanase enzyme concentration and exposure time (**C**).

**Figure 2:**
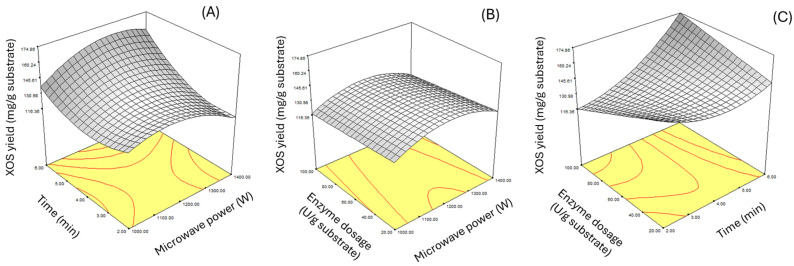
Response surface plot of xylooligosaccharide production from barley malt residue after 12 h enzyme incubation, showing the relationship between microwave power and exposure time (**A**), microwave power and xylanase enzyme concentration (**B**), and xylanase enzyme concentration and exposure time (**C**).

When Equations (1) and (2) were used to predict the maximum response values using the Design-Expert 6.0.10. program, it was found that for enzyme incubations of 4 and 12 h, using a microwave power of 1235.1 W, an exposure time of 6 min, and a xylanase enzyme concentration of 89.12 U/g substrate gave the highest yield of XOS of 208.05 and 172.61 mg/g substrate, respectively. Equations (1) and (2) were used to predict the maximum response value using the Design-Expert 6.0.10 program, it was displayed as a response surface plot, as shown in Figure 1 and Figure 2.

There were remarkable changes in the contents of hemicellulose, especially in the microwave-pretreated BMR. Microwave-assisted BMR hemicellulose extraction was more efficient than conventional heating since microwaves emitted energy uniformly in the material, reducing process time and enhancing efficiency and homogeneity [17,51]. This pretreatment led to fragmentation and swelling, resulting in lignin and hemicellulose degradation and consequently increasing the yield of pentoses [52]. According to the previous report by Ethaib et al. [53], some parameters greatly influenced the microwave pretreatment efficiency of biomass, including the use of biomass loads, microwave powers, and exposure time. Moreover, microwave-assisted enzymatic hydrolysis offers improvement of energy efficiency through faster heating rates and lower energy consumption, while reducing use and waste impact [54].

**Figure 3:**
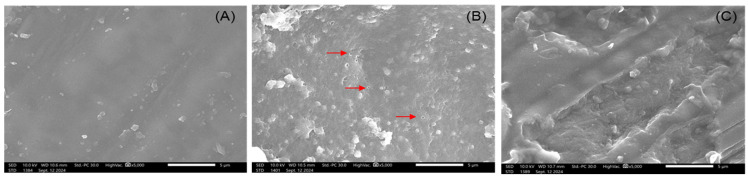
Scanning electron microscopy (SEM) analysis of the surface of raw barley malt residue (**A**); microwave-pretreated barley malt residue (**B**); and microwave-assisted enzymatic hydrolysis of barley malt residue (**C**). The porous structure is indicated by the red arrows.

SEM analysis also revealed the surface morphology of various BMR samples. Untreated BMR was rigid, having a smooth surface without pores (Figure 3A). The surface structure of raw BMR mainly consisted of cellulose, hemicellulose, and lignin, with a complex lignocellulosic structure that limited enzyme penetration. The BMR after microwave treatment (Figure 3B) developed a porous structure due to thermal fragmentation and swelling leading to the degradation of lignin and hemicellulose [55]. This pretreatment increased enzymatic hydrolysis through lignin removal selectively without altering carbohydrates, thereby increasing porosity as well as surface area [56]. Binod et al. [57] reported that microwave pretreatment was enough to obtain high degradation of the cell wall, which increased the surface area of BMR towards enzyme contact. It partially de-crystallized the BMR and brought about the breakage of the rigid structure. The porosity of BMR increased after 24 h of enzymatic hydrolysis, as shown in Figure 3C. The amorphous hemicellulose structure was degraded to extract XOS and other sugars by enzymes, whereas cellulose remained intact in the rough BMR.

### 3.3. Purification of Xylooligosaccharides

**Figure 4:**
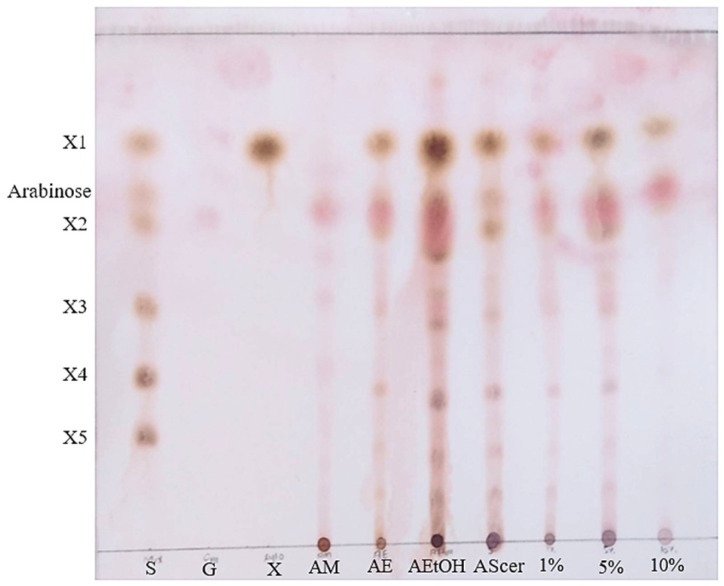
TLC chromatogram of BMR in comparison to the sugar components after treatments. S: standard mixture of glucose (G) and xylose (X); AM: microwave treatment; AE, enzyme hydrolysis; AEtOH, ethanol treatment; AScer: *Saccharomyces cerevisiae* treatment; 5%: 5% activated carbon treatment; 10%: 10% activated carbon treatment.

After enzymatic hydrolysis, the supernatant was collected by centrifugation at 12,000× *g* for 10 min. To remove water-soluble high-molecular-weight impurities, ethanol precipitation, activated carbon adsorption (at concentrations of 1, 5, and 10% by weight), and *Saccharomyces cerevisiae* (yeast) treatments were employed. As shown in Figure 4, the purification steps successfully separated the high-molecular components. The oligosaccharides produced were identified as DP (X1-X5), arabinose, and glucose. TLC results for the treatment of *S. cerevisiae* reveal a distinct band containing XOS, where glucose was used as a carbon source. Glucose might be the product generated from the starch-containing polysaccharide in BMR, by hydrolysis activated by heat during microwave treatment [58]. Ethanol treatment revealed the presence of xylose and X2-X5 along with trace amounts of glucose. Moreover, 10% of activated carbon treatments demonstrated only xylose, arabinose, and glucose. The results show that the *S. cerevisiae* and activated carbon adsorption method was better than the ethanol precipitation method, which was found in earlier literature [35]. Microbial treatment has been recently recognized as an efficient method for monosaccharide removal with less effect on other compounds [59]. In contrast to the physical and chemical methods including ethanol precipitation and activated carbon adsorption, the target oligosaccharides were separated into monosaccharides with non-specific reactions resulting in the loss of target oligosaccharides [60]. According to the characteristics of environmentally friendly minimal processing, fewer chemicals were used from viable yeast treatment compared with ethanol precipitation, and activated carbon adsorption and *S. cerevisiae* treatment was selected for further experiments.

### 3.4. Structural Characterization of XOS

**Figure 5:**
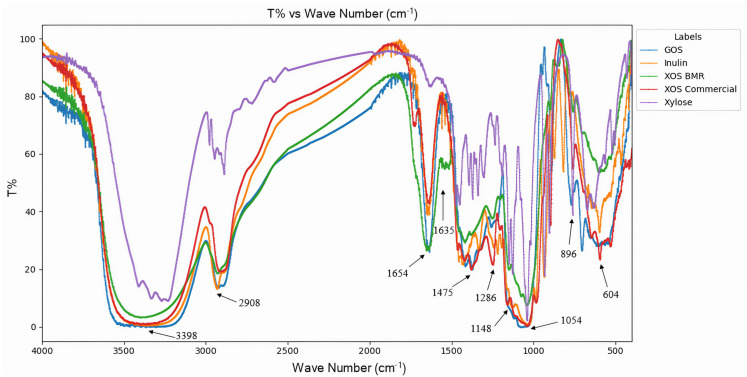
FTIR spectra of BMR-XOS, commercial-XOS, inulin, GOS, and xylose.

FTIR spectroscopy was used to identify the functional groups, purity, structure, and intermolecular interactions of purified oligosaccharides, as shown in Figure 5. The spectra of BMR-XOS, commercial XOS, GOS, inulin, and xylose were compared. The spectrum manifested the typical transmittance bands of hemicellulosic oligosaccharides with peak assignments based on literature reports. The typical peaks around 3398, 2908, 1654, 1475, 1286, 1148, 1054, and 896 cm^−1^ were almost identical with commercial XOS. The same band pattern in XOS was reported earlier [61,62]. The peak around 3398 cm^−1^ usually corresponds to the vibration of the -OH group. The C–H stretching peak at 2908 cm^−1^ can be a sign of the integrity and quality of the oligosaccharides. The peak around 1635 cm^−1^ represents the CH streaking of XOS [62]. Narrow bands of 1654 and 1475 cm^−1^ were attributed to the CH, OH, or CO stretching and bending vibrations of hemicelluloses, while the 896 cm^−1^ peak indicated the 1-4 β configuration of xylan [63]. A peak at 1286 cm^−1^ indicated C=O and C-O stretching [62], while a small vibration at 1054 cm^−1^ suggested the presence of 4-O-methylglucuronoxylan-type oligosaccharides and polymers [64]. The peak around 604 cm^−1^ was identified as the stretching vibration of the C-O-C bond and some C-C bonds in XOS [61].

### 3.5. Growth Promotion of Probiotics by BMR-XOS

A prebiotic is a selectively fermented ingredient that causes a specific change in the composition and/or activity of the gastrointestinal microbiota resulting in confirmed health benefits. Criteria should be well defined for categorizing a food ingredient as a prebiotic. In this study, *L. lactis* TISTR 1401, *L. brevis* FS 2.1, and *L. casei* TISTR 1463 were able to utilize BMR-XOS for cell growth, based on an increase in viable cell counts on a modified MRS medium. Maximum viable cell counts (logCFU/mL) and maximum specific growth rates (μ_max_) in modified MRS supplemented with various carbon sources are shown in Table 6. All probiotics demonstrated a greater capacity to grow in MRS media supplemented with xylose compared with glucose. Among the commercial prebiotics, the highest viable cell counts for *L. brevis* FS 2.1, and *L. lactis* TISTR 1401 were observed in MRS supplemented with commercial XOS (10.18 and 10.36 logCFU/mL, respectively), while, *L. casei* TISTR 1463 achieved its maximum viable cell with commercial GOS (10.31 logCFU/mL). Interestingly, BMR-XOS significantly enhanced the growth rate of *L. lactis* TISTR 1401, *L. brevis* FS 2.1, and *L. casei* TISTR 1463, achieving maximum specific growth rates (μ_max_) of 1.085, 0.891 and 0.532 h^−1^, respectively, surpassing those observed with commercial XOS. Based on the previous report, *L. lactis* TISTR 1401 was recognized as xylose-utilizing lactic acid bacteria [65], and *L. brevis* FS 2.1 was also shown to be xylooligosaccharide-fermenting probiotic lactic acid bacteria [37]. Meanwhile, *L. casei* TISTR 1463 was recognized as GOS and FOS utilizing probiotic bacteria. Considering the μ_max_ values (Table 5), the high and low values obtained from each carbon source represented the preferable carbon source, even though the maximum viable cells reached nearly the same value. For example, the maximum viable cell of *L. casei* TISTR 1463 on commercial XOS carbon source was almost the same value as those of *L. lactis* TISTR 1401 and *L. brevis* FS 2.1, but its μ_max_ value was approximately 10 times lower when compared with others. This elucidates the suitable carbon source for each microbe. Additionally, in MRS medium supplemented with BMR-XOS, the pH decreased from an initial value of approximately 6.5 to final values of 4.72, 4.82, and 4.64 for *L. lactis* TISTR 1401, *L. brevis* FS 2.1, and *L. casei* TISTR 1463, respectively, after 48 h of fermentation, confirming the growth of all probiotic strains. The varying efficiencies in utilizing BMR-XOS may be attributed to the specific oligosaccharide utilization mechanisms unique to each strain [66]. This study confirms that BMR-XOS served as a suitable carbon source for some probiotic strains and can be used as an alternative prebiotic ingredient. However, the evaluation of the efficacy and possibility in large-scale production of BMR-XOS requires further investigation.

## 4. Conclusions

This study illustrates that BMR is an agricultural waste that could be used as a source of natural xylan for the production of xylooligosaccharides (XOS). BMR-XOS showed the most promising results, supporting rapid growth and efficient degradation and utilization of XOS by the selected probiotic strains. The observed fermentation activity, including an increase in optical density (OD), a gradual decrease in pH, and effective XOS utilization, suggests that BMR-XOS-fermenting probiotics could be viable candidates for use as probiotics. These findings highlight the potential of BMR-XOS-fermenting probiotic strains as effective prebiotics. Additionally, the study also explored the feasibility of microwave-assisted enzymatic hydrolysis of BMR, which improved XOS yield and facilitated the removal of impurities from the final product. The xylanase treatment followed by the biological purification process by a generally recognized as safe (GRAS) microbe such as *S. cerevisiae* demonstrated the potential to achieve food-grade XOS, marking a significant step toward large-scale commercial production. With these advancements, we anticipate the commercial production of XOS from BMR as a sustainable and cost-effective prebiotic source. Besides, among the recent global threats from environmental problems, as well as the vigorous expansion of non-communicable diseases (NCDs) in human beings, this research also demonstrates a promising contribution to the development of sustainable, health-promoting prebiotics from BMR, addressing both waste management and efficient green processing in the food industry while also aligning with sustainability goals.

## Figures and Tables

**Table 1:** Factors and levels specified in the Box–Behnken design experiment.

Parameters		Levels	
	−1	0	1
Microwave power (Watts), A	1000	1200	1400
Exposure time (min), B	2	4	6
Enzyme dosage (U/g substrate), C	20	60	100

**Table 2:** Box–Behnken design for determining the optimum conditions for the production of XOS.

Run	Microwave Power (Watts), A	Exposure Time (min), B	Enzyme Dosage (U/g Substrate), C
1	1000	2	60
2	1400	2	60
3	1000	6	60
4	1400	6	60
5	1000	4	20
6	1400	4	20
7	1000	4	100
8	1400	4	100
9	1200	2	20
10	1200	6	20
11	1200	2	100
12	1200	6	100
13	1200	4	60
14	1200	4	60
15	1200	4	60
16	1200	4	60
17	1200	4	60

Experiments were performed in triplicate, corresponding to the central point.

**Table 3:** Chemical composition of barley malt residue.

Composition	Content (%, *w*/*w*)	Composition	Content (%, *w*/*w*)
Crude fiber	32.05 ± 0.15	Holocellulose	38.17 ± 1.12
Carbohydrate	37.04 ± 0.13	Cellulose	20.77 ± 0.31
Protein	20.64 ± 0.14	Hemicellulose	17.40 ± 0.37
Fat	4.00 ± 0.16	Lignin	14.50 ± 1.05
Moisture content	3.74 ± 0.24		
Total ash	2.50 ± 0.18		

Note: Values are expressed as mean ± SD, n = 3.

**Table 4:** Amount of XOS produced in each experimental set.

Run	A Microwave Power (Watts)	B Exposure Time (min)	C Enzyme Dosage (U/g Substrate)	Yield of XOS (mg/g Substrate)	
				4 h	12 h
1	1000	2	60	154.4 ± 0.6	142.33 ± 0.8
2	1400	2	60	118.56 ± 0.7	107.09 ± 0.1
3	1000	6	60	180.8 ± 0.1	145.13 ± 0.2
4	1400	6	60	180.8 ± 0.9	145.13 ± 0.3
5	1000	4	20	121.48 ± 0.1	113.84 ± 0.9
6	1400	4	20	117.88 ± 0.5	137.91 ± 0.7
7	1000	4	100	111.17 ± 0.2	108.18 ± 0.6
8	1400	4	100	99.32 ± 0.3	130.43 ± 0.8
9	1200	2	20	137.67 ± 0.3	155.2 ± 0.4
10	1200	6	20	163.87 ± 0.9	152.58 ± 0.3
11	1200	2	100	118.94 ± 0.3	113.28 ± 0.1
12	1200	6	100	212.14 ± 0.3	176.8 ± 0.2
13	1200	4	60	164.47 ± 0.2	135.09 ± 0.4
14	1200	4	60	167.02 ± 0.1	139.22 ± 0.2
15	1200	4	60	164.81 ± 0.7	135.55 ± 0.6
16	1200	4	60	160.42 ± 0.3	135.15 ± 0.9
17	1200	4	60	163.54 ± 0.7	138.76 ± 0.9

Note: Values are expressed as mean ± SD, n = 3.

**Table 5:** Analysis of variance (ANOVA) results for factors affecting the amount of XOS from barley malt residue.

Variable	4 h		12 h	
	Coefficient Estimate	*p*-Value	Coefficient Estimate	*p*-Value
Model/Intercept	164.05	0.0005 sig	136.75	0.0493 sig
A—Microwave power	−6.41	0.0953	1.39	0.7401
B—Exposure time	26.01	0.0001	12.72	0.0157
C—Enzyme dosage	0.084	0.9806	−3.85	0.3686
A^2^	−25.55	0.0008	−14.35	0.0356
B^2^	20.14	0.0032	12.52	0.0580
C^2^	−26.04	0.0008	0.19	0.9735
AB	8.96	0.0985	8.81	0.1644
AC	−2.06	0.6743	−0.45	0.9383
BC	16.75	0.0092	16.54	0.0225
R-Squared (R^2^)	0.9587		0.8262	
Adjusted R-Squared	0.9056		0.6028	

**Table 6:** Maximum viable cell counts, maximum specific growth rate, and final pH of *L. lactis* TISTR 1401, *L. brevis* FS 2.1, and *L. casei* TISTR 1463, cultivated in modified MRS medium supplemented with different carbon sources.

Carbon Sources	*L. lactis* TISTR 1401			*L. brevis* FS 2.1			*L. casei* TISTR 1463		
	Max. Viable Cell (logCFU/mL)	μ_max_ (h^−1^)	Final pH at 48 h	Max. Viable Cell (logCFU/mL)	μ_max_ (h^−1^)	Final pH at 48 h	Max. Viable Cell (logCFU/mL)	μ_max_ (h^−1^)	Final pH at 48 h
Glucose	9.26 ± 0.17 ^a^	1.006	4.19	8.88 ± 0.05 ^b^	0.731	4.08	9.13 ± 0.01 ^ab^	0.368	4.15
Xylose	10.43 ± 0.01 ^a^	1.222	4.37	10.39 ± 0.01 ^b^	0.870	4.41	9.39 ± 0.02 ^c^	0.145	4.38
Commercial XOS	10.36 ± 0.03 ^a^	0.993	4.39	10.18 ± 0.01 ^b^	0.791	4.43	9.42 ± 0.01 ^c^	0.403	4.79
Commercial GOS	9.39 ± 0.01 ^b^	0.955	5.24	8.37 ± 0.01 ^c^	0.551	4.75	10.31 ± 0.02 ^a^	0.510	4.74
Commercial Inulin	9.40 ± 0.01 ^a^	0.406	5.31	9.37 ± 0.01 ^b^	0.813	3.79	9.41 ± 0.01 ^a^	1.114	3.72
BMR-XOS	10.38 ± 0.01 ^a^	1.085	4.72	10.31 ± 0.01 ^b^	0.891	4.82	10.36 ± 0.01 ^a^	0.532	4.64

Note: Means in rows with different superscripts are statistically different at *p* < 0.05.

## Data Availability

The original contributions presented in the study are included in the article; further inquiries can be directed to the corresponding author.

## Supplementary Materials

The following supporting information can be downloaded at https://www.mdpi.com/article/10.3390/plants14050769/s1. Figure S1: Amount of total sugar (a) and reducing sugar (b) produced in each experimental set.
