# Supplementary material for: Xylooligosaccharides from Barley Malt Residue Produced by Microwave-Assisted Enzymatic Hydrolysis and Their Potential Uses as Prebiotics
Source: Plants (Basel). 2025 Mar 3;14(5):769. doi: 10.3390/plants14050769 (PMC11901517; doi:10.3390/plants14050769)
Supplement: Supplementary file 1 [file plants-14-00769-s001.zip › plants-3426595-supplementary.pdf]

**Supplementary Materials:** The following are available online at www.mdpi.com/xxx/s1,

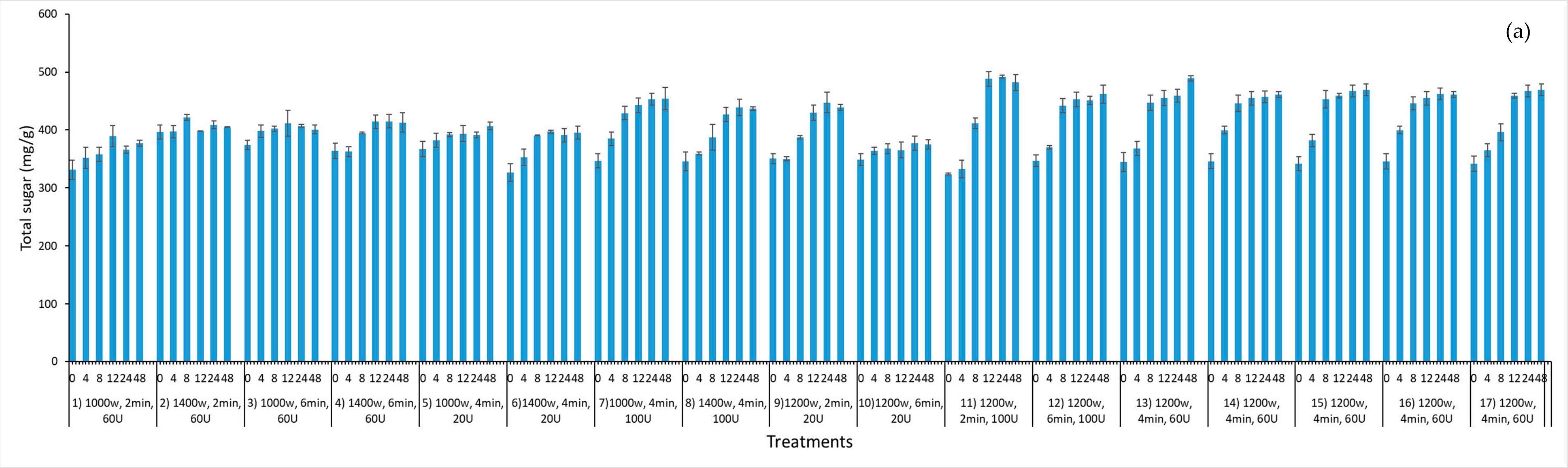


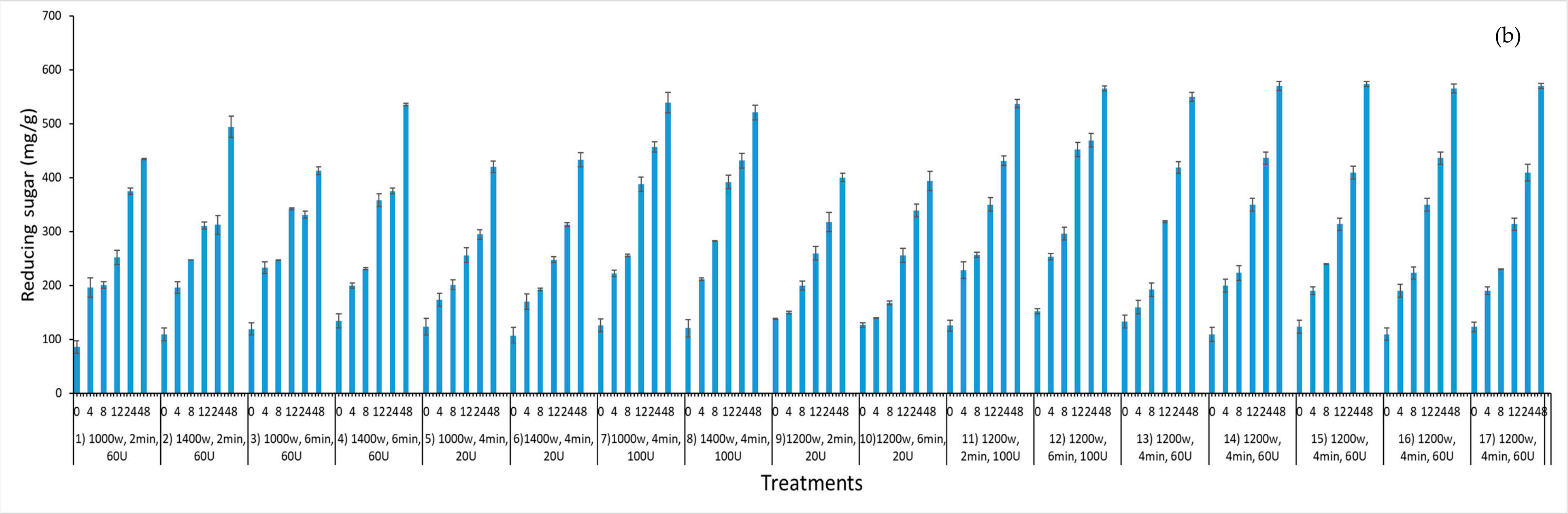


**Figure S1.** Amount of total sugar (a) and reducing sugar (b) produced in each experimental set.
